# SCM-198 Ameliorates Cognitive Deficits, Promotes Neuronal Survival and Enhances CREB/BDNF/TrkB Signaling without Affecting Aβ Burden in AβPP/PS1 Mice

**DOI:** 10.3390/ijms160818544

**Published:** 2015-08-07

**Authors:** Zhen-Yi Hong, Shuang-Shuang Yu, Zhi-Jun Wang, Yi-Zhun Zhu

**Affiliations:** Shanghai Key Laboratory of Bioactive Small Molecules, Department of Pharmacology, School of Pharmacy, Fudan University, Shanghai 201203, China; E-Mails: cerulian@163.com (Z.-Y.H.); yss2359450@163.com (S.-S.Y.); oscarwzj@gmail.com (Z.-J.W.)

**Keywords:** SCM-198, amyloid-β, Alzheimer’s disease, Morris water maze, novel object recognition, cAMP-responsive element-binding protein, brain-derived neurotrophic factor, tropomyosin-related kinase B

## Abstract

SCM-198 is an alkaloid found only in *Herba leonuri* and it has been reported to possess considerable neuroprotective effects in animal models of ischemic stroke, Parkinson’s disease and Alzheimer’s disease (AD). In this study, we demonstrated for the first time that 3-month oral SCM-198 treatment could significantly improve both recognition and spatial memory, inhibit microgliosis and promote neuronal survival in amyloid-β protein precursor and presenilin-1(AβPP/PS1) double-transgenic mice without affecting amyloid-β (Aβ) burden. In addition, decreases in cAMP-response element-binding protein (CREB) phosphorylation, brain-derived neurotrophic factor (BDNF) and tropomyosin-related kinase B (TrkB) phosphorylation were attenuated by SCM-198 both *in vivo* and in primary cortical neurons, which could be blocked by protein kinase A (PKA) inhibitors, suggesting the involvement of upstream PKA in enhancing the BDNF/TrkB/CREB signaling by SCM-198. Our results indicate that SCM-198, a drug that could promote neuronal survival and enhance BDNF/TrkB/CREB signaling, has beneficial effects on behavioral and biochemical alterations without affecting Aβ burden in AβPP/PS1 mice and might become a potential drug candidate for AD treatment in the future.

## 1. Introduction

Alzheimer’ disease (AD), the most prevalent neurodegenerative disorder, is a complex disease in which many factors are involved. Clinically its characteristic symptom is gradual decline in memory and cognition in AD patients, while pathologically it is characterized by amyloid-β (Aβ) deposition, neurofibrillary tangles, neuronal loss and neuroinflammation [1].

Over the past 100 years, only four cholinesterase inhibitors and one *N*-methyl-d-aspartate (NMDA)-receptor antagonist have been officially approved for AD treatment. These drugs are capable of improving symptoms like cognitive deficits and global function, but unable to substantially stop the progression of AD [2]. Currently, the most prevailing hypothesis of AD is the Aβ cascade model and a large amount of research is now devoting a great deal of attention to developing various methods or drugs for intervention with Aβ production or clearance. However, several most recent phase Ⅲ clinical trials yielded disappointing results suggesting that patients treated with Aβ-lowering agents did not show any significant improvements in cognition [3,4,5], indicating that therapeutic strategies targeting Aβ might need further investigations and modifications. Many studies also showed that cognitive deficits could be alleviated without altering Aβ burden [6,7]. Despite these negative results from clinical trials targeting Aβ, the neurotoxicity of Aβ is confirmed and now widely accepted. Evidence showed that Aβ could alter hippocampal-dependent synaptic plasticity by inhibiting cAMP-response element-binding protein (CREB) phosphorylation, resulting in reduced long-term potentiation (LTP). CREB is required for the formation of long-term memory and decrease in CREB phosphorylation was reported to precede morphological alterations in AD models [8,9,10]. Brain-derived neurotrophic factor (BDNF) is one of the target genes of CREB, and the decline of BDNF/ tropomyosin-related kinase B (TrkB) signaling, which plays critical roles in neuronal survival and synaptic plasticity has been previously reported. Pharmacologically or technically enhancing CREB/BDNF/TrkB/signaling have been reported to be feasible for AD treatment [11,12].

SCM-198 (chemical name 4-guanidino-*n*-butyl syringate) is an active phenolic alkaloid found in *Herba leonuri* [13]. In addition to its outstanding cardioprotective properties, SCM-198 has recently been explored for the treatment of ischemic stroke, AD and Parkinson’s disease in Sprague-Dawley (SD) rats. The main therapeutic mechanisms of action involved are inhibition of oxidative stress, mitochondrial protection and alleviation of neuroinflammation [14,15,16]. Our previous study was conducted in Aβ_40_-injected SD rats, which is an acute model for assessing the anti-neuroinflammatory and the cognition-improving activities of SCM-198 [16]. In this study, we examined the possible neuroprotective effects of SCM-198 in amyloid-β protein precursor and presenilin-1 (AβPP/PS1) double-transgenic mice and demonstrated for the first time that long-term oral SCM-198 treatment enhanced cognitive performance, inhibited microglial overactivation and neuronal apoptosis in AβPP/PS1 transgenic mice without altering Aβ burden. More importantly, for the first time, we showed that SCM-198 enhanced CREB/BDNF/TrkB/signaling both *in vitro* and *in vivo* which could be blocked by protein kinase A (PKA) inhibitors (H89 or Rp-cAMPS), suggesting the involvement of PKA in the protection of AβPP/PS1 mice by SCM-198. Taken together, our data indicate that SCM-198 could be a potential therapeutic drug for AD treatment in the future.

## 2. Results

### 2.1. SCM-198 Rescued Recognition Memory Deficits in AβPP/PS1 Mice in NOR Test

**Figure 1:**
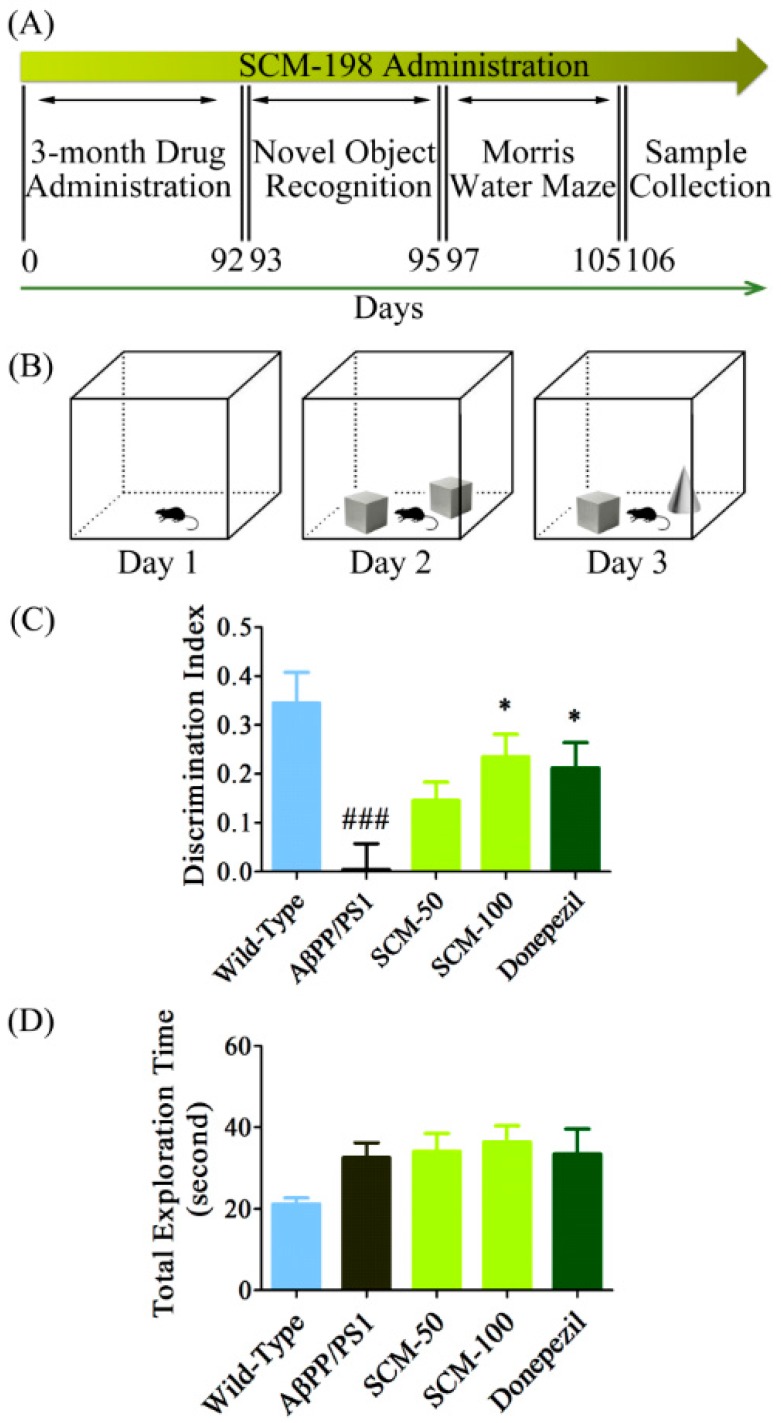
SCM-198 rescued recognition memory deficits in AβPP/PS1 mice in novel object recognition (NOR) test. All mice began receiving different treatments at 6 months of age and were fed continuously for 3 months. 9-month old mice were then tested in NOR test. (**A**) Schematic timeline of SCM-198 treatment and behavioral evaluations; (**B**) Schematic illustration of NOR test; (**C**) Discrimination index (DI) of AβPP/PS1 mice on the 3^rd^ day of NOR test; (**D**) Total exploration time of AβPP/PS1 mice on the 3^rd^ day of NOR test (SCM: SCM-198; DON: donepezil). Data represent mean ± SEM of 10–11 mice per group. * *p* < 0.05, Tukey’s test *vs.* AβPP/PS1 group; ### *p* < 0.001, Tukey’s test *vs.* wild-type group.

After 3-month administration of SCM-198 and DON, no significant differences in body weight were observed among the experimental groups (data not shown). We first assessed the effects of SCM-198 on cognitive deficits in AβPP/PS1 mice. The NOR test, which assesses the cognitive performances that rely on the activities of the frontal cortex and hippocampus, is based on the rodent’s innate preference for novel objects over familiar ones [17,18]. During the retention phase (Day 3), vehicle-treated AβPP/PS1 mice displayed significantly less interest in novel object compared with that of wild-type mice with an average DI of 0.0046, indicating the difficulty for vehicle-treated AβPP/PS1 mice in differentiating between novel and familiar objects. By contrast, 50 mg/kg SCM-198-, 100 mg/kg SCM-198- or DON-treated AβPP/PS1 mice tended to spend more time exploring the novel object with average DIs of 0.1462, 0.2349 and 0.2128, respectively. Significant improvements were found in 100 mg/kg SCM-198- and DON-treated groups (*F* (4, 47) = 6.333, *p* = 0.0004, Figure 1C), indicating the neuroprotective effects of SCM-198 in ameliorating cognitive impairment of AβPP/PS1 mice. A slight decrease in total exploration time was observed in wild-type group, but no significant differences were found among the five groups (*F* (4, 47) = 1.932, *p* = 0.1207, Figure 1D).

### 2.2. SCM-198 Alleviated Spatial Memory Deficits in AβPP/PS1 Mice in MWM Test

**Figure 2:**
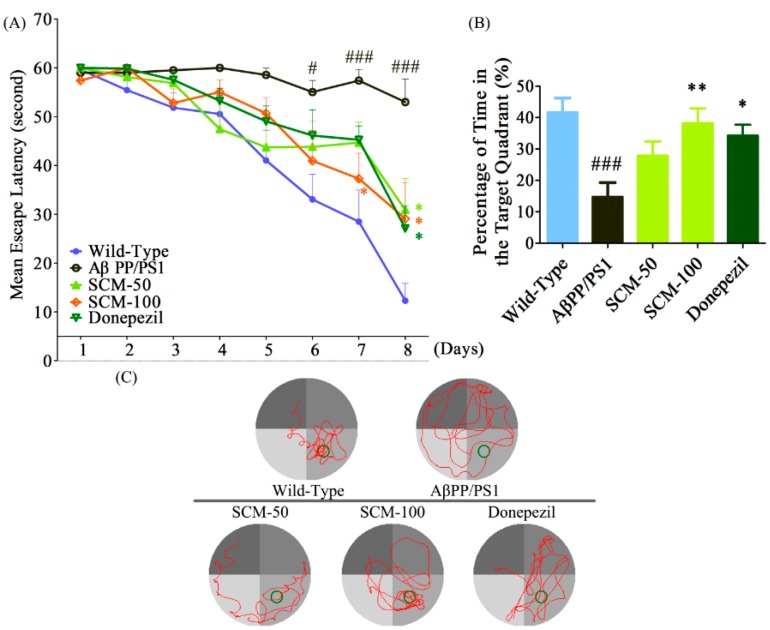
SCM-198 alleviated spatial memory deficits in AβPP/PS1 mice in the Morris water maze (MWM) test. All mice began receiving different treatments at 6 months of age and were fed continuously for 3 months. 2 days after NOR test, 9-month old mice were tested in MWM test. (**A**) Mean escape latency during the training phase from day 1 to day 8; (**B**) Percentage of time spent in the target quadrant during the probe trial on the 9th day; (**C**) Representative swimming paths of AβPP/PS1 mice during the probe trial on 9th day of MWM test (SCM: SCM-198; DON: donepezil; Green ring means target area). Data represent mean ± SEM of 10–11 mice per group. * *p* < 0.05, ** *p* < 0.01, Tukey’s test *vs.* AβPP/PS1 group; # *p* < 0.05, ### *p* < 0.001, Tukey’s test *vs.* wild-type group.

Two days after NOR test, animals were subjected to MWM test for the evaluation of spatial memory. During the acquisition phase (Day 1 to Day 8), mean escape latency (time for reaching the invisible platform) for all experimental groups became progressively shorter. No significant differences were observed from Day 1 to Day 5, (Day 1, *F* (4, 47) = 0.9881, *p* = 0.4233; Day 2, *F* (4, 47) = 1.220, *p* = 0.3149; Day 3, *F* (4, 47) = 2.237, *p* = 0.0793; Day 4, *F* (4, 47) = 2.237, *p* = 0.0793, respectively, Day 5, *F* (4, 47) = 2.563, *p* = 0.0505, Figure 2A). Statistically significant differences emerged on Day 6 (Day 6, *F* (4, 47) = 2.607, *p* = 0.0475, Figure 2A) and significant therapeutic effects of SCM-198 and DON were observed on the last two training days, 100 mg/kg SCM-198-treated mice showed considerable improvements in cognition (Day 7, *F* (4, 47) = 5.939, *p* = 0.0006; Day 8, *F* (4, 47) = 7.121, *p* = 0.0001, respectively, Figure 2A). Two-way repeated-measures ANOVA analysis showed an extremely significant drug effect (*F* (4, 329) = 8.571, *p* < 0.0001) and time effect (*F* (4, 329) = 8.571, *p* < 0.0001). During the probe trial, all mice were swimming in the pool without the escape platform. The percentage of time spent in the target quadrant was applied to evaluate the memory retention of animals. Vehicle-treated AβPP/PS1 mice spent significantly less time in the target quadrant compared with wild-type mice, indicating the significant cognitive impairment in this group. 100 mg/kg SCM-198 treatment produced significant increase in percentage of time in the target quadrant (*F* (4, 47) = 5.977, *p* = 0.0006, Figure 2B,C). No statistically significant differences in swimming speed were observed among groups.

### 2.3. Long-Term SCM-198 Treatment Did Not Affect Aβ Burden

**Figure 3:**
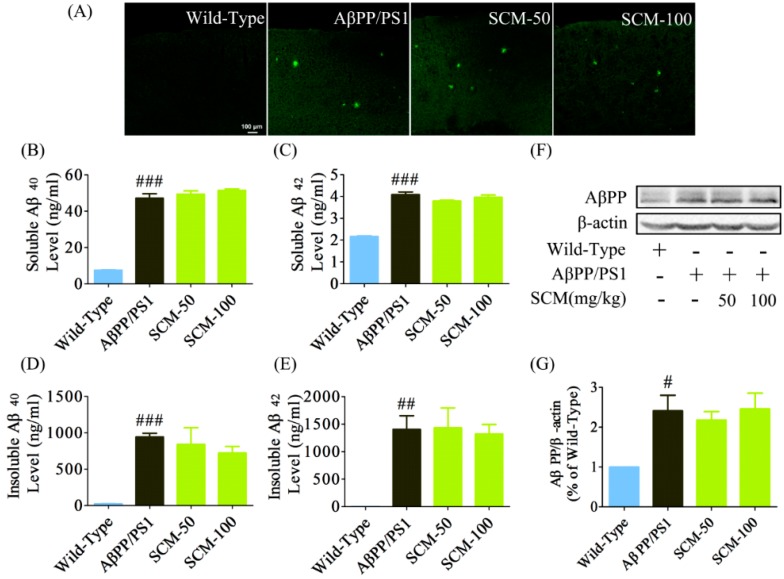
Long-term SCM-198 treatment did not affect amyloid-β (Aβ) burden. Wild-type and AβPP/PS1 mice began receiving different treatments at 6 months of age and were fed continuously for 3 months. All mice were sacrificed after the MWM test and brain slices were stained with rabbit polyclonal anti-Aβ primary antibody and were observed using a confocal microscope. (**A**) Examples of cortical Aβ staining in AβPP/PS1 mice (green; scale bar, 100 μm; *n* = 6 mice/group); Cortical soluble Aβ_40_ (**B**), soluble Aβ_42_ (**C**), insoluble Aβ_40_ (**D**) and insoluble Aβ_42_ (**E**) levels were measured using ELISA kits (*n* = 6 mice/group); (**F**,**G**) amyloid-β protein precursor (AβPP) expression was analyzed by Western blot (*n* = 6 mice/group) (SCM: SCM-198). Data represent mean ± SEM. # *p* < 0.05, ## *p* < 0.01, ### *p* < 0.001, Tukey’s test *vs.* wild-type group.

Both fluorescent IHC and ELISA methods were applied to investigate whether SCM-198 could decrease Aβ burden in AβPP/PS1 mice brain. Although significant cognitive improvements were observed in NOR and MWM tests, no decrease in Aβ burden was observed after long-term SCM-198 treatment (Figure 3A). Data from ELISA assay showed that significant differences in Aβ_40_ and Aβ_42_ levels (*F* (3, 20) = 175.10, *p* < 0.0001; *F* (3, 20) = 108.80, *p* < 0.0001, respectively, Figure 3B,C) or insoluble Aβ_40_ and Aβ_42_ levels (*F* (3, 20) = 10.92, *p* = 0.0002; *F* (3, 20) = 8.571, *p* = 0.0007, respectively, Figure 3D,E) were only found between the wild-type group and AβPP/PS1 group, and post-hoc analysis showed no differences between SCM-198-treated groups and AβPP/PS1 group. We also analyzed AβPP protein expression by Western blot. Compared with wild-type group, significant increase of AβPP protein expression was observed in AβPP group and SCM-198-treated groups (*F* (3, 20) = 5.333, *p* = 0.0073, Figure 3F,G). However, post-hoc analysis also showed no difference in AβPP expression between the AβPP group and SCM-198-treated groups. Overall, SCM-198 has no effects on Aβ load in AβPP/PS1 mouse brain.

### 2.4. SCM-198 Alleviated Excessive Microglial Activation and Decreased TNF-α Level in AβPP/PS1 Mice

**Figure 4:**
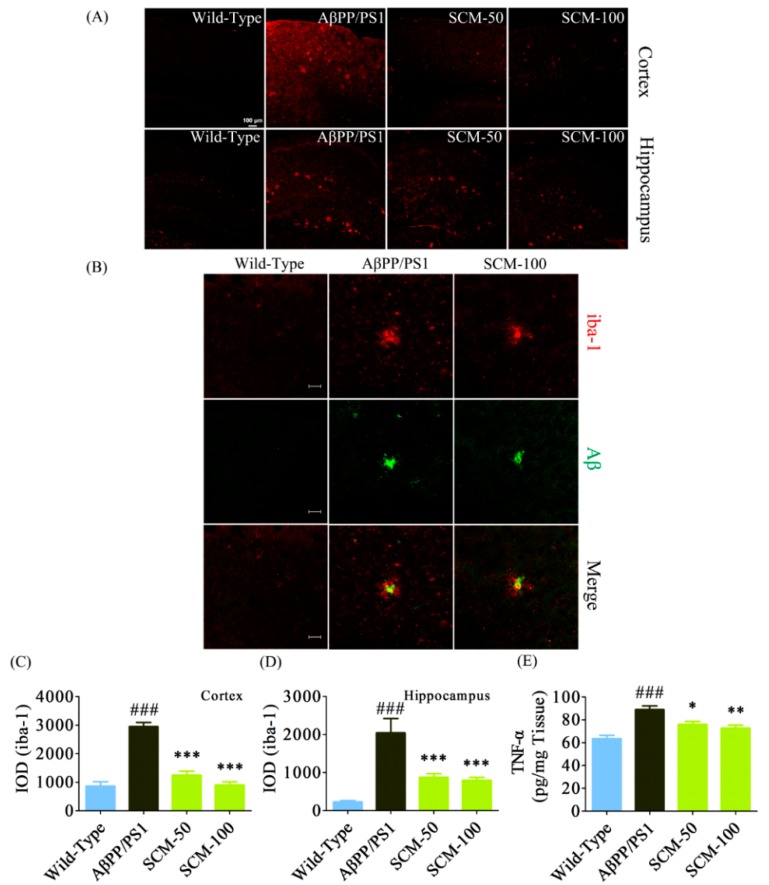
SCM-198 alleviated excessive microglial activation and decreased TNF-α levels in AβPP/PS1 mice. Wild-type and AβPP/PS1 mice began receiving different treatments at 6 months of age and were fed continuously for 3 months. Microgliosis was visualized by fluorescent immunostaining after behavioral tests. (**A**) Cortical (**upper** panel) and hippocampal (**lower** panel) microglia were stained using goat polyclonal anti-iba-1antibody (red; scale bar, 100 μm) and were observed using a confocal microscope; (**B**) Representative confocal images of microglia (iba-1; red) and senile plaques (Aβ; green) in 9-month-old AβPP/PS1 mice (scale bar, 20 μm); (**C**) Integrated optical density (IOD) of cortical iba-1 staining (*n* = 6 mice/group); (**D**) IOD of hippocampal iba-1staining (*n* = 6 mice/group); (**E**) Cortical tumor necrosis factor-α (TNF-α) was measured with commercial ELISA kits (*n* = 6 mice/group). Data represent mean ± SEM. * *p* < 0.05, ** *p* < 0.01, *** *p* < 0.001, Tukey’s test *vs.* AβPP/PS1 group; ### *p* < 0.001, Tukey’s test *vs.* wild-type group.

As excessive chronic neuroinflammation is now considered one of the major risk factors in the development of AD, we assessed microglial activation in both cortex and hippocampus of AβPP/PS1 mice. As can be seen in Figure 4A, marked increases in microgliosis in both cortex and hippocampus were observed in vehicle-treated AβPP/PS1 mice as compared with those of wild-type mice. SCM-198 at doses of 50 and 100 mg/kg significantly reduced microglial overactivation in the cortex (*F* (3, 116) = 52.65, *p* < 0.0001, Figure 4C) and hippocampus (*F* (3, 116) = 15.07, *p* < 0.0001, Figure 4D). Aggregation and recruitment of microglial cells with large cellular bodies around the amyloid plaques were observed in both vehicle- and SCM-198-treated AβPP/PS1 mice. Strong immunoreactivity of iba-1 is still evident in microglial cells that are far from the plaques in vehicle-treated AβPP/PS1 mice, whereas this strong iba-1 immunoreactivity was less pronounced in SCM-198-treated group (Figure 4B). ELISA assay showed that cortical TNF-α, one of the proinflammatory cytokines released by overactivated microglia, significantly increased in vehicle-treated AβPP/PS1 mice, while 50 and 100 mg/kg SCM-198 prevented TNF-α elevation in a dose-dependent manner (*F* (3, 20) = 13.23, *p* < 0.0001, Figure 4E), which was consistent with the results of immunostaining of microglia.

### 2.5. SCM-198 Promoted Neuronal Survival both in Vitro and in Vivo

**Figure 5:**
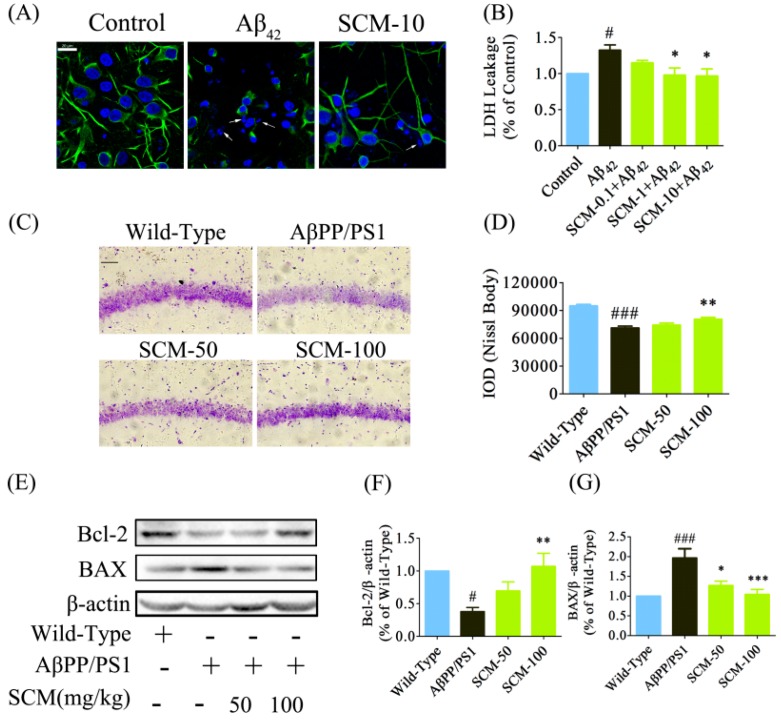
SCM-198 promoted neuronal survival both *in vitro* and *in vivo*. Primary cortical neurons were pretreated with or without 10 μM SCM-198 for 2 h followed by 24 h stimulation of 20 μM Aβ_42_. Supernatant was collected for LDH leakage measurement and neurons were stained using polyclonal rabbit anti-MAP2 antibody. (**A**) SCM-198 prevented neuronal apoptosis and protected neurons against neurite breakage (green: MAP2; blue: DAPI; arrows showed nuclear fragmentation, scale bar, 20 μm); (**B**) SCM-198 dose-dependently decreased LDH leakage in primary cortical neurons. Wild-type and AβPP/PS1 mice began receiving different treatments at 6 months of age and were fed continuously for 3 months; (**C**) Representative images of Nissl staining in CA1 region of AβPP/PS1 mice (scale bar, 200 μm); (**D**) Quantification of Nissl staining in CA1 region of AβPP/PS1 mice (*n* = 6 mice/group); Bcl-2 (**E**,**F**) and BAX (**E**,**G**) protein expressions were analyzed by Western blot (*n* = 6–7 mice/group) (SCM: SCM-198). Data represent mean ± SEM. * *p* < 0.05, ** *p* < 0.01, *** *p* < 0.001, Tukey’s test *vs.* AβPP/PS1 group or Aβ_42_-treated group; # *p* < 0.05, ### *p* < 0.001, Tukey’s test *vs.* wild-type group or control group.

Cortical neurons were pretreated with or without 10 μM SCM-198 for 2 h followed by 24 h stimulation with 20 μM Aβ_42_. No toxicity was observed in primary neurons for SCM-198 within the tested concentrations (data not shown). SCM-198 could prevent neuronal apoptosis and protect the neurons against neurite breakage (Figure 5A) and LDH leakage (*F* (4, 25) = 4.585, *p* = 0.0065, Figure 5B) induced by Aβ_42_ stimulation. Nissl staining was used to evaluate neuronal degeneration in AβPP/PS1 mice. About 25% of neuronal loss in the CA1 region was observed in vehicle-treated AβPP/PS1 mice compared with wild-type mice, while only 15.3% of neuronal loss was observed in 100 mg/kg SCM-198 -treated group, indicating the significant protective effect of SCM-198 against neuronal loss (Figure 5C, *F* (3, 116) = 29.51, *p* < 0.0001, Figure 5D). Bcl-2 and BAX proteins are important members of Bcl-2 family which play important roles in regulating neuronal apoptosis in CNS. SCM-198 was found to dose-dependently up-regulate the anti-apoptotic Bcl-2 (*F* (3, 20) = 6.333, *p* = 0.0034, Figure 5E,F) protein and down-regulated the pro-apoptotic BAX protein (*F* (3, 24) = 9.774, *p* = 0.0002, Figure 5E,G) in AβPP/PS1 mice.

### 2.6. SCM-198 Enhanced CREB/BDNF/TrkB Signaling both In Vivo and In Vitro

**Figure 6:**
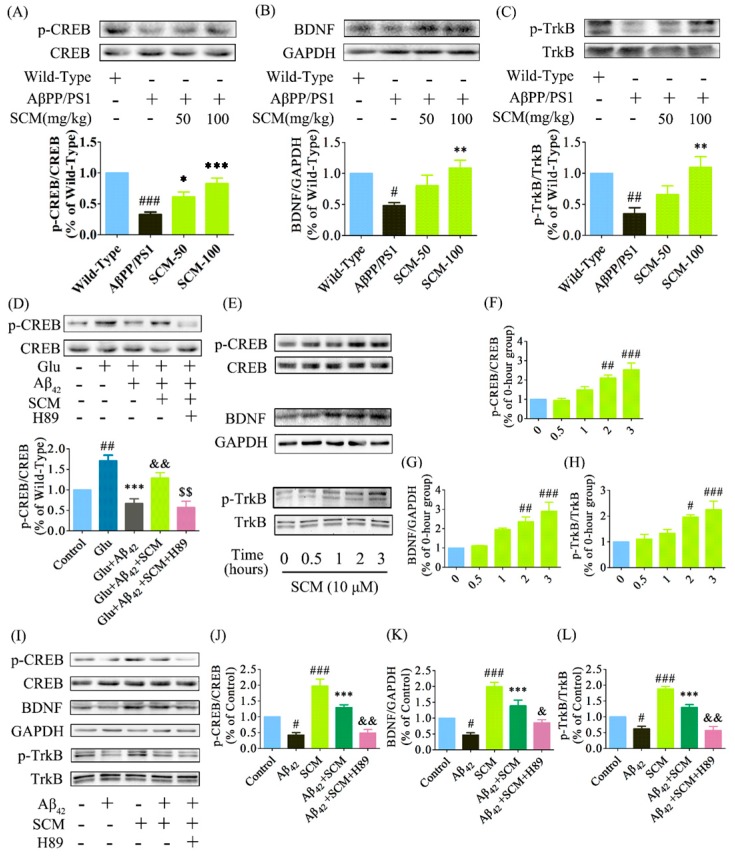
SCM-198 enhanced CREB/BDNF/TrkB signalling both *in vivo* and *in vitro*. Wild-type and AβPP/PS1 mice began receiving different treatments at 6 months of age and were fed continuously for 3 months. Proteins from hippocampus of AβPP/PS1 mice were analyzed by Western blot using antibodies against (**A**) p-CREB and total CREB (*n* = 6 mice/group); (**B**) BDNF and GAPDH (*n* = 6 mice/group) and (**C**) p-TrkB and total TrkB (*n* = 5mice/group) (SCM: SCM-198). Data represent mean ± SEM. * *p* < 0.05, ** *p* < 0.01, *** *p* < 0.001, Tukey’s test *vs.* AβPP/PS1 group; # *p* < 0.05, ## *p* < 0.01, ### *p* < 0.001, Tukey’s test *vs.* wild-type group. Primary cortical neurons were pretreated with or without 10 μM SCM-198 or 50 μM H89 (inhibitor of protein kinase A) for 1 h, followed by stimulation with 10 μM Aβ_42_ for 1 h; (**D**) After 10-min stimulation with 100 μM glutamate (Glu), proteins were extracted and analyzed by Western blot using antibodies against p-CREB and total CREB. Data represent mean ± SEM of more than four independent experiments. *** *p* < 0.001, Tukey’s test *vs.* only Glu-treated group; ## *p* < 0.01, Tukey’s test *vs.* control group; && *p* < 0.01. Tukey’s test *vs.* Glu + Aβ_42_-treated-group; $$ *p* < 0.01, Tukey’s test *vs.* Glu + Aβ_42_ + SCM-treated-group; SCM-198 at 10 μM time-dependently increased p-CREB (**E**,**F**), BDNF (**E**,**G**) and p-TrkB (**E**,**H**) expressions in primary neurons. Neurons were then pretreated with or without 10 μM SCM-198 or 50 μM H89 (inhibitor of protein kinase A) for 2 h, followed by 24-h exposure to 10 μM Aβ_42_. SCM-198 significantly inhibited the reductions in p-CREB (**I**,**J**), BDNF (**I**,**K**) and p-TrkB (**I**,**L**) expressions induced by Aβ_42_ exposure, which could be blocked by H89 treatment. Data represent mean ± SEM of more than four independent experiments. ***p < 0.001, Tukey’s test *vs.* only Aβ_42_-treated group; # *p* < 0.05, ## *p* < 0.01, ### *p* < 0.001, Tukey’s test *vs.* control group; & *p* < 0.05, && *p* < 0.01, Tukey’s test *vs.* SCM + Aβ_42_-treated-group.

**Figure 7:**
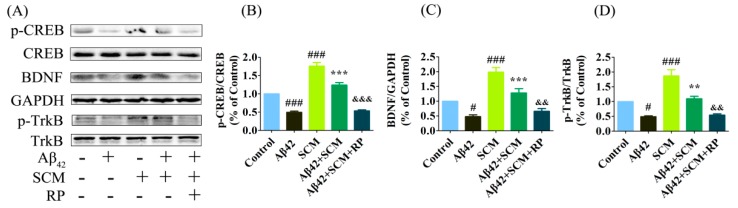
SCM-198 enhanced CREB/BDNF/TrkB signalling in primary cortical neurons. Primary cortical neurons were pretreated with or without 10 μM SCM-198 or 50 μM Rp-cAMPS (RP, inhibitor of protein kinase A) for 2 h, followed by 24 h exposure to 10μM Aβ_42_. SCM-198 significantly inhibited the reductions in p-CREB (**A**,**B**), BDNF (**A**,**C**) and p-TrkB (**A**,**D**) expressions induced by Aβ_42_ exposure, which could be blocked by RP-cAMPS treatment. Data represent mean ± SEM of five independent experiments. ** *p* < 0.01, *** *p* < 0.001, Tukey’s test *vs.* only Aβ_42_-treated group; # *p* < 0.05, ### *p* < 0.001, Tukey’s test *vs.* control group; && *p* < 0.01, &&& *p* < 0.001, Tukey’s test *vs.* SCM + Aβ_42_-treated-group.

Memory decline is one of the important features of AD. Multiple lines of evidence have shown that deficits in long-term potentiation and synaptic transmission occur very early and precede massive Aβ deposition and behavioral abnormalities. The CREB protein, the phosphorylation of which is significantly decreased in both AD transgenic animal models and human AD post-mortem brains, plays a key role in memory formation [10,19,20,21]. Western blot analysis of CREB phosphorylation (p-CREB) showed a reduction by approximately 60% in the hippocampus of vehicle-treated AβPP/PS1 mice as compared with that of wild-type mice, while this reduction was dose-dependently increased by long-term SCM-198 treatment and was reversed back to wild-type levels in 100 mg/kg SCM-198-treated AβPP/PS1 group (*F* (3, 20) = 14.45, *p* < 0.0001, Figure 6A). Significant declines in BDNF and TrkB phosphorylation (p-TrkB) were also observed in vehicle-treated AβPP/PS1 group and 100 mg/kg SCM-198 significantly enhanced the BDNF (*F* (3, 20) = 6.228, *p* = 0.0037, Figure 6B) and p-TrkB levels (*F* (3, 16) = 7.919, *p* = 0.0018, Figure 6C) in AβPP/PS1 mice. No significant changes were detected in total CREB or TrkB levels. Glutamate was first used to quickly induce the elevation of p-CREB levels *in vitro*. In primary cortical neurons, p-CREB was strongly induced by 10-min incubation with 100 μM glutamate, while 1-h treatment of 10 μM Aβ_42_ decreased p-CREB levels. 1-h pretreatment of 10 μM SCM-198 significantly alleviated Aβ_42_-induced down-regulation of p-CREB, which could be completely blocked by 50 μM H89 (*F* (4, 30) = 15.87, *p* < 0.0001, Figure 7D). No significant changes were observed in total CREB levels. 10 μM SCM-198 itself could also time-dependently increased p-CREB (*F* (4, 25) = 14.03, *p* < 0.0001, Figure 6E,F), BDNF (*F* (4, 15) = 11.79, *p* = 0.0002, Figure 6E,G) and p-TrkB (*F* (4, 20) = 9.076, *p*= 0.0002, Figure 6E,H) levels in primary cortical neurons and significant increases were observed after 2 h treatment with SCM-198. Compared with control group, 24 h stimulation with 10 μM Aβ_42_ caused significant reductions in p-CREB, BDNF and p-TrkB levels, which were reversed by 2-h pretreatment with 10 μM SCM-198 in neurons and this protective effect could also be blocked by 50 μM H89 or 50 μM Rp-cAMPS. In conclusion, these results suggested that SCM-198 could enhance CREB/BDNF/TrkB signaling in AβPP/PS1 mice and prevent Aβ_42_-induced reductions in p-CREB (*F* (4, 20) = 28.23, *p* < 0.0001, Figure 6I,J; *F* (4, 20) = 87.46, *p* < 0.0001, Figure 7A,B), BDNF (*F* (4, 25) = 25.55, *p* < 0.0001, Figure 6I,K; *F* (4, 20) = 30.86, *p* < 0.0001, Figure 7A,C) and p-TrkB (*F* (4, 20) = 41.74, *p* < 0.0001, Figure 6I,L; *F* (4, 20) = 28.72, *p* < 0.0001, Figure 7A,D) levels in cortical neurons. Besides, the compound itself could increase p-CREB, BDNF and p-TrkB expressions, indicating that SCM-198 could promote neurotrophic signaling, at least partially, via regulating the PKA-CREB pathway.

## 3. Discussion

In this study, effects of long-term SCM-198 treatment on cognitive functions were evaluated in AβPP/PS1 transgenic mice. Treatments were started at the 6th month of age when AβPP/PS1 mice already showed apparent Aβ accumulation and significant impairments inbasal synaptic transmission and LTP [19]. Cognition-improving effects of SCM-198, with no change in Aβ burden, were significant in this transgenic model and our data showed for the first time that SCM-198 enhanced CREB/BDNF/TrkB signaling both in primary neurons and in AβPP/PS1 mice, which could be blocked by PKA inhibitors, indicating that SCM-198 could promote BDNF/TrkB neurotrophic signaling via, at least partially, through regulating the upstream PKA-CREB pathway.

Two commonly used behavioral methods, NOR and MWM tests, were applied for evaluations of non-spatial recognition memory and spatial reference memory, respectively. The NOR task depends less on the integrity of hippocampus and is not stressful to the animals, while the MWM task largely depends on intact hippocampal functions [22]. Significant improvements were observed in SCM-198-treated and DON-treated groups in both behavioral paradigms with 100 mg/kg as the optimal dose, indicating that long-term administration of SCM-198 improved both non-spatial and spatial memory in AβPP/PS1 mice. Our preliminary experiment showed that doses higher than 100 mg/kg could not further improve cognitive performances in AβPP/PS1 mice, thus 100 mg/kg could be considered as the optimal dose of SCM-198 for improving cognitive performances of AβPP/PS1 mice.

Pathologically, brains from AD patients are characterized by intracellular accumulation of hyperphosphorylated tau protein and extracellular senile plaques which are mainly composed of Aβ peptides of 39–43 amino acid residues. Under normal conditions, AβPP is mainly cleaved by α-secretase within the Aβ domain through the non-amyloidogenic pathway, yielding soluble sAβPPα and preventing Aβ production. In AD patients’ brains, the amyloidogenic pathway is significantly enhanced and AβPP undergoes cleavage by β- and γ-secretases, releasing soluble sAβPPβ and Aβ peptides (mainly Aβ_40_ and Aβ_42_). Much evidence has shown that Aβ_40_ is more abundantly produced than Aβ_42_, while Aβ_42_ is more neurotoxic and more amyloidogenic [7,23,24]. However, anti-amyloid treatments still remain controversial today. Many previous studies have shown negative results in human clinical trials: (1) Semagacestat, a small-molecule γ-secretase inhibitor, significantly reduced Aβ_40_ and Aβ_42_ levels in AD patients’ brains, but failed to ameliorate patients’ cognitive impairment [3]; (2) Solanezumab, a monoclonal antibody that promoting Aβ clearance in central nervous system (CNS), was demonstrated to be ineffective in improving cognitive performance of patients with mild to moderate AD [4]; and (3) Bapineuzumab, another humanized monoclonal antibody that capable of binding to different forms of Aβ, also failed to show efficacy in a phase Ⅲ clinical trial involving patients with mild-to-moderate AD [5]. Also, there was evidence that some therapeutic methods improved cognitive performance of transgenic mice without affecting Aβ burden: (1) Pyrrolidine dithiocarbamate improved spatial learning by regulating AKT-glycogen synthase kinase 3 signaling pathway in AβPP/PS1 mice without reducing Aβ load [6]; (2) no linear correlation was found between the worsening of memory function and the brain Aβ deposits, and some studies demonstrated that cognitive decline preceded plaque Aβ deposition [25]. Therefore, although the toxicity of Aβ oligomers or plaques has already been confirmed by many studies and is widely accepted today, it is most likely that Aβ and the plaques do not play as big a role as previously thought or anti-Aβ therapeutics may be more effective if given during the very early or even asymptomatic stage of AD. From another perspective, cognition improving interventions without affecting Aβ burden might also be feasible for AD patients. Our study showed the inability of SCM-198 to reduce AβPP expression or brain Aβ burden, indicating that the compound shows considerable neuroprotective and cognition-improving effects, but not in an Aβ-reducing way.

In agreement with our previous study in Aβ_40_-injected SD rats, we found that SCM-198 also significantly inhibited microglial overactivation in AβPP/PS1 mouse brain, further confirming its anti-neuroinflammatory properties. Similar extents of recruitment of microglia to the senile plaques were observed in both vehicle-treated AβPP/PS1group and SCM-198-treated group, but fewer activated microglia were found in the areas at a distance from the plaques in SCM-198-treated group. Since our compound does not affect Aβ burden and could decrease proinflammatory cytokines such as TNF-α in the mouse brain, we hypothesize that SCM-198 may not affect the microglial cells around the plaques, but may rather inhibit the activated microglial cells remote from the plaques. DON is well-known for its narrow therapeutic window. According to Pfizer’s material safety data sheet, the median lethal dose of DON by oral administration is estimated to be 45.2 mg/kg in mice and 32.6 mg/kg in rats. As to SCM-198, the maximum tolerated dose by oral route is >5 g/kg, indicating its high safety margin. One point worth noting is that significant microglial inhibition could be achieved by either 50 or 100 mg/kg SCM-198, while only high dose treatment could significantly improve cognitive performance in AβPP/PS1 mice. Possible explanations are: (1) the mechanism of inhibiting microglia might be essential but insufficient alone to improve the cognition of AβPP/PS1 mice and it must work synergistically with other mechanisms to achieve the qualitative changes in cognition; (2) inhibition of microglial cells remote from the senile plaques may provide limited contributions to cognitive improvements. Since SCM-198 has no effects on Aβ burden and microglial cells around the plaques, we guess that drug properties affecting the microglia around the plaques might be more critical for cognitive improvements. As we have reported in our previous study, SCM-198 could inhibit overactivated microglia via JNK and NF-κB pathways, and further studies are needed to confirm whether SCM-198’s effect on these pathways in microglia contribute to the improvement of cognition in AD transgenic mouse models. Also, studies on tau phosphorylation by SCM-198 are needed and important to further evaluate the overall effects of this new compound in AD treatment.

CREB phosphorylation is critical for the formation of hippocampally-dependent long-term memory. Impaired cyclic AMP (cAMP)/PKA signaling and decreased p-CREB were observed in the brains of AD patients [26], whereas treatments that increase CREB activity could alleviate the spatial memory deficits in AD transgenic mice [20]. Glutamate could stimulate *N*-methyl-d-aspartic acid (NMDA) receptors, elevate intracellular calcium and subsequently increase p-CREB level and was first used in our study to quickly elevate p-CREB in primary neurons [27]. Our data showed that Aβ_42_ at 10 μM did not cause neuronal death (data not shown), but led to a significant decrease in p-CREB, which was activated by incubation with 100 μM glutamate. This *in vitro* data was consistent with *in vivo* results that p-CREB significantly decreased in vehicle-treated AβPP/PS1 mice. SCM-198 significantly reversed p-CREB reductions both *in vitro* and *in vivo*, which was abolished by PKA inhibitors, H89 or Rp-cAMPS. BDNF, one of the downstream target genes of CREB, plays important roles in supporting neuronal survival and facilitating both early and late phase of LTP, and therefore enhances synaptic transmission and synaptogenesis in CNS [12,28,29,30]. BDNF is the high affinity ligand for TrkB receptor and it exerts its biological functions via phosphorylating TrkB receptor [31]. Much evidence has accumulated indicating that BDNF-TrkB signaling declines in AD patients and in patients with mild cognitive impairment, and this deficient signaling may even precede the decline of choline acetyltransferase (ChAT) activity [32,33]. Our data showed that 100 mg/kg SCM-198 significantly increased BDNF and p-TrkB levels in the mouse brain and inhibited the decreased BDNF and p-TrkB expressions in primary neurons induced by 24 h exposure to 10 μM Aβ_42_. This positive effect on BDNF-TrkB signaling was also blocked by H89 or Rp-cAMPS treatment, indicating that SCM-198 exerts neuroprotective effects at least partly through regulating the upstream PKA-CREB signaling pathway. Further studies are necessary to explore whether SCM-198 plays a role in regulating LTP and synaptic transmission, and several other animal AD models are needed to further confirm and investigate the therapeutic effects and other underlying mechanisms of action of SCM-198.

## 4. Experimental Section

### 4.1. Regents

Bovine serum albumin (BSA) was purchased from Amresco (Solon, OH, USA). Poly-d-lysine and 4ʹ-6-diamidino-2-phenylindole (DAPI) were from Sigma-Aldrich (St. Louis, MO, USA). PKA inhibitor H89 was from Melonepharma (Dalian, China) and another PKA inhibitor Rp-cAMPS was purchased from Santa Cruz (Santa Cruz Biotechnology, Santa Cruz, CA, USA). Donepezil (DON) hydrochloride, with purity greater than 99%, was from Energy Chemical (Shanghai, China). SCM-198 (purity ≥ 99%), was synthesized according to our previous study [15]. Fetal bovine serum and cell culture medium were purchased from Gibco (Grand Island, NY, USA).

### 4.2. Animals and Drug Administration

Sixty-two male AβPP/PS1 (strain name: AβPP/PSN (B6); Tg [AβPPswe, PSEN1dE9] 85Dbo) double-transgenic mice and their littermate wild-type mice from Model Animal Research Center (Nanjing, China) were used in this study (13 mice for wild-type group, vehicle-treated AβPP/PS1group, SCM-198-treated groups, 10 mice for DON group). Animals were kept in a specific pathogen-free and 12 h light/dark cycled environment with constant humidity (around 50%) and temperature (22 ± 1 °C). Food and distilled water were available *ad libitum*. Drugs or saline was administered by gavage once daily for 3 months (from 6 to 9 months of age) until the end of the behavioral tests. This is based on the previous study that obvious Aβ plaques could be observed when these mice reach six months of age and significant cognitive impairment could be detected at around seven months of age [19,34,35,36]. Animals were randomly divided into five groups (10 to 11 mice per group): wild-type group, vehicle-treated AβPP/PS1 group (both treated with 0.9% saline containing 1% (*w*/*v*) sodium carboxymethylcellulose, 50 and 100 mg/kg SCM-198-treated groups, 4.0 mg/kg DON-treated group (positive control).The range of doses of SCM-198 were selected according to our previous study in SD rats and our preliminary data indicated that doses higher than 100 mg/kg did not further improve cognitive performances in AβPP/PS1 mice. The dose of donepezil was determined according to previous published findings with AD transgenic mouse models or old mice [36,37,38]. Both compounds were administered 1 h before the behavior tests. Body weight was recorded weekly until the end of the behavioral tests. All procedures were approved by the Animal Ethics Committee of Fudan University and were in accordance with the guidelines of Regulations of Experimental Animal Administration of People’s Republic of China (14 November 1988).

### 4.3. Novel Object Recognition (NOR) Test

Fifty-two mice were used for behavioral testing (10 mice for wild-type group, 11 mice for vehicle-treated AβPP/PS1 group, 11 mice for 50 mg/kg SCM-198-treated group, 10 mice for 100 mg/kg SCM-198-treated group, 10 mice for DON group). Behavioral tests were conducted in a room with constant temperature (23 ± 1 °C) and humidity (around 50%) and NOR task was performed when the animals were 9 months of age. A three-day protocol was employed: on the 1st day, mice were placed in the middle of an empty white Plexiglas box (40 cm × 40 cm × 40 cm) and were free to explore the entire box for 10 min; on the 2nd day, mice were allowed to explore the entire box for 10 min in the presence of two identical objects (object A, the familiar object); on the 3rd day, one object A was replaced with a novel object (object B, the novel object) and mice were placed again into the box and allowed to explore for 10 min (Figure 1A,B). The time spent sniffing, licking or physically touching the objects while facing them was defined as exploration time, while leaning against or standing on the object was not considered exploratory behavior. Total exploration time and discrimination index (DI) were recorded and calculated as follows: total exploration time = exploration time (novel) + exploration time (familiar); DI = [exploration time (novel) − exploration time (familiar)]/total exploration time × 100%. The designation of novel object (A or B) and the position of the novel object (left or right) were counterbalanced within each group to reduce possible bias due to location or object preference [17,18].

### 4.4. Morris Water Maze (MWM) Test

Forty eight hours after the NOR test, animals were subjected to the MWM test for 9 consecutive days to evaluate spatial learning and memory behavior (Figure 1A). The MWM apparatus consists of a black Plexiglas circular pool (120 cm in diameter), a transparent platform (20–35 cm in height, 6 cm in diameter) and a video tracking system. The pool was equally divided into four quadrants and filled with clean water (21 ± 1 °C) which was made opaque with a non-toxic white paint (Dyestuffs Research Institute, Shanghai, China). Spatial cues of different shapes and colors were attached to the walls surrounding the pool. During the acquisition phase (from day 1 to day 8), the platform was located in the middle of one quadrant (target quadrant) and was submerged 1 cm below the water surface. Each mouse was given two platform trials per day to locate the invisible platform randomly from 2 starting points which were equidistant from the platform. Mice that failed to find the platform within 60 s were gently directed onto it and left there for 15 s. The value of daily escape latency for each mouse was defined as the mean of two trials. On the 9th day (probe trial), each mouse was placed into the water from a new starting point and allowed to navigate for 60 s in the pool in which the platform was withdrawn. Percentage of time in the target quadrant and swimming speed were recorded and calculated using the Doctor Mice software (Mobile datum, Shanghai, China) [16,25].

### 4.5. Quantification of Aβ_40_ and Aβ_42_ by Enzyme-Linked Immunosorbent Assay (ELISA)

Cortical Aβ_40_ and Aβ_42_ levels of AβPP/PS1 mice were quantified using solid phase sandwich ELISA kits (IBL, Fujioka, Japan). Aβ extraction from brain tissue was performed according to a previously published method with minor modifications [39]. Briefly, the cortex was homogenized in protease inhibitor-containing Tris-buffered saline (TBS+) (20 mM Tris-HCl, 150 mM NaCl; pH 7.6; 0.8 mL TBS buffer per 200 mg tissue) and centrifuged at 100,000× *g* for 60 min at 4 °C in a ultracentrifuge (CP100MX, Hitachi, Tokyo, Japan). The supernatant was collected and kept at −80 °C for TBS-soluble Aβ measurement. The pellet was washed with the same TBS+ buffer and centrifuged in the same way as the previous step before being resuspended in 6 M guanidine-HCl buffer (in 50 mM Tris buffer, pH 7.6; 0.5 mL 6 M guanidine-HCl buffer per 50 mg tissue). After brief sonication, the pellet-containing guanidine-HCl buffer was kept at room temperature (RT) for 30 min, followed by centrifuged at 100,000× *g* for 30 min at 4 °C. The supernatant was collected and kept for TBS-insoluble Aβ measurement. Both TBS+-soluble and TBS+-insoluble Aβ-containing supernatants were subjected to ELISA according to the manufacturer’s protocol.

### 4.6. Western Blot Analysis

Protein extraction from primary neuron cultures or mouse hippocampus and western blot analysis were done according to previously published methods [16]. The primary antibodies applied were: polyclonal rabbit anti-AβPP, monoclonal rabbit anti-phospho-CREB (Ser 133), monoclonal rabbit anti-CREB, polyclonal rabbit anti-Bcl-2, polyclonal rabbit anti-BAX, monoclonal rabbit anti-β-actin, monocolonal rabbit anti-GAPDH (1:1000, Cell Signaling, Danvers, MA, USA), polyclonal rabbit anti-phospho-TrkB (Thy 706), polyclonal rabbit anti-TrkB (1:1000, Bioworld Technology, St. Louis Park, MN, USA), polyclonal rabbit anti-BDNF (1:1000, Proteintech Group Inc., Chicago, IL, USA). Horseradish peroxidase conjugated secondary antibodies (1:5000) were purchased from Santa Cruz (Santa Cruz Biotechnology, Santa Cruz, CA, USA). Proteins were detected using Immobilon™ Western Chemiluminescent HRP Substrate (Millipore, Temecula, CA, USA) in a Bio-Rad ChemiDoc™ XRS+ System (Bio-Rad, Hercules, CA, USA) and were analyzed by ImageJ software (NIH, Bethesda, MD, USA).

### 4.7. Fluorescent Immunohistochemistry (IHC) and Nissl Staining

AβPP/PS1 mice were anesthetized with 7% chloral hydrate (0.5 mL per 100 g body weight) and transcardially perfused with 50 mL of 0.9% saline, followed by 50–100 mL of ice-cold 4% paraformaldehyde (PFA) in 0.1 M phosphate buffer (PB). After 72-h post-fixation in 4% PFA (in 0.1 M PB) and gradient dehydration in 20%–30% sucrose (in 0.1 M PB) at 4 °C, brains were serially cut into 20-μm-thick coronal sections using a cryostat microtome (Thermo Fisher Scientific, Waltham, MA, USA). After extensive washing with 0.01 M phosphate buffered saline (PBS) (pH 7.4), sections were immersed in 0.3% Triton X-100 for 15–30 min and then transferred to blocking solution (10% BSA and 0.1% NaN_3_) for 1 h at RT. After incubation with goat polyclonal anti-iba-1antibody (1:500, Abcam, Cambridge, MA, USA) or rabbit polyclonal anti-Aβ primary antibody (1:200, Abcam, Cambridge, MA, USA) overnight at 4 °C, sections were washed with 0.01 M PBS and subsequently incubated with Alexa Fluor 488-conjugated donkey anti-rabbit or Alexa Fluor 594-conjugated donkey anti-goat secondary antibody (1:200, Molecular Probes, Eugene, OR, USA). Brain sections were mounted onto clean microscope slides with Fluoromount™ (Sigma-Aldrich, St. Louis, MO, USA) and were observed using a Carl Zeiss LSM710 confocal microscopy (Carl Zeiss, Inc., Jena, Germany). For Nissl staining, brain sections were stained in cressyl violet solution (Beyotime, Shanghai, China) for 5 min, rinsed in distilled water, dehydrated in 95% ethanol, cleared in xylene, mounted with neutral balsam (Sinopharm Chemical Reagent Co., Ltd., Shanghai, China) and photographed by a light microscope (Carl Zeiss Inc., Thornwood, NY, USA). There are reports showing that the dorsal hippocampus and parietal cortex of the mouse brain have been reported to play critical roles in spatial information processing and atrophy in hippocampus and parietal cortex can be found in patients with early AD [40,41]; therefore, brain sections between −1.34 and −2.30 mm relative to bregma were kept and analyzed for Iba-1 expression and Nissl bodies. For each animal, every ninth section was collected and an average of 5 sections was analyzed. All micrographs were taken under the same conditions and quantified using ImagePro Plus 6.0 software (Media Cybernetics, Silver Spring, MD, USA) and the same minimum threshold value was set for every photo for the purpose of background correction. Data were expressed as integrated optical density (IOD), which is equal to the area × average density of the hippocampus occupied by immunoreactivity and represented as the mean ± SEM.

### 4.8. Measurements of Tumor Necrosis Factor-α (TNF-α) and Lactate Dehydrogenase (LDH) Activity

LDH activity was measured using a commercial kit (Jiancheng Bioengineering, Nanjing, China) according to manufacturer’s instructions. For primary neurons, cell supernatant was collected after different treatments for measurement of LDH leakage. TNF-α levels were quantified using a commercial ELISA kit (Boatman Biotech, Shanghai, China). The absorbance was recorded at 450 nm for both LDH activity assay and TNF-α measurement. Protein concentration was measured using a BCA-100 Protein Quantitative Analysis kit (Shenergy Biocolor, Shanghai, China).

### 4.9. Aβ_42_ Preparation, Primary Cortical Neuron Culture and in Vitro Drug Treatment

A stock solution of Aβ_42_ (1 mg/mL) (ChinaPeptides, Shanghai, China) was prepared by first dissolving Aβ_42_ peptides into sterilized distilled water, followed by dilution with PBS (calcium-free) solution. The stock solution was then incubated for 4 days at 37 °C before its application in primary neurons. Cortical neuron cultures from embryos of SD rats (17–18 days of gestation) were prepared as previously reported [16] and were maintained for 10–14 days before treatments. Neurons were pretreated with or without 10 μM SCM-198 or 50 μM H89 for 1 h and then stimulated with 10 μM Aβ_42_ for 1 h. After 10 min stimulation with 100 μM glutamate, cells were lysed and analyzed by Western blot. For fluorescent staining, neurons were pretreated with or without 10 μM SCM-198 for 2 h followed by stimulation with 20 μM Aβ_42_ for 24 h. Cells were then fixed with 4% PFA, blocked with 10% BSA for 1 h at RT and stained with polyclonal anti-MAP2 primary antibody (1:50, Cell Signaling, Danvers, MA, USA) and Alexa Fluor 488-conjugated donkey anti-rabbit secondary antibody. 1 µg/mL DAPI was used for nuclear staining. Neurons were observed using a Carl Zeiss LSM710 confocal microscopy (Carl Zeiss, Inc., Jena, Germany).

### 4.10. Statistical Analysis

Statistical analyses were performed using GraphPad Prism (La Jolla, CA, USA). Data were presented as mean ± SEM. Multi-group comparisons were performed by one-way analysis of variance (ANOVA) or two-way repeated-measures ANOVA, followed by Tukey’s *post-hoc* analysis. *p*-values less than 0.05 were considered to be statistically significant.

## 5. Conclusions

Taken collectively, this is the first time that SCM-198 has been demonstrated to alleviate cognitive deficits in AβPP/PS1 mice.SCM-198 inhibits microglial overactivation, enhances CREB/BDNF-TrkB signaling and therefore promotes neuronal survival both *in vitro* and *in vivo*, suggesting that SCM-198 might become a promising candidate drug for AD therapy.
